# The “Help” Question—A Plea for Bridget

**Published:** 1870-01

**Authors:** Ben H. Pratt


### THE "HELP" QUESTION--A PLEA FOR BRIDGET.

By BEN H. PRATT, A. M.

"Yes, but wherein lies your theme's appropriateness to a Medical Journal?" triumphantly cries my too critical friend, peering over my shoulder as I note down my heading.

To the well meaning objector let me put the following questions.

In the wide range of subjects now dazing the public eye and the public mind, in all manner of prints, from the flickering weekly of an obscure hamlet to the blazing daily of the metropolis—two editions and a triple sheet—what question comes closer to the heart and home, the business and bosom of every man, woman and child, than this same "Help Question?"—Whom does it *not* affect? If you, however, dear reader mine, can truthfully say that you have no part nor lot in this matter, I would place no thorn in the pillow of so envied a mortal. This paper is not for you, Fortunatissima! Pass on to the next article.

But few there are, raised by fortune or a competence from off the lower shelf of society, to whom this subject is not only interesting, but vitally important; and as *The Bistoury* is nothing if not domestic, so domestic a matter surely cannot err on the side of inappropriateness in the columns of a journal so professedly home-influencing.

Having prefaced my subject thus inadequately to some—unnecessarily to others—let us step directly into the merits of the question by an unbidden visit to a coterie of mistresses of households, and per consequence, rulers of maidservants known as "help," maids-of-all-work, girls, &c., &c.

A tea-party may suffice for this—or a quilting—or a formal call—or a knitting circle—or a neighborly happen in—for you must know that no assembly of woman kind, dignified by what title soever, is above discussing the "Help Question." If not first or second in order of topics, it is sure to take the third place—having but two rivals worthy of its mettle, viz. dress, and neighborhood gossip.

Not to allow the interesting discussion, (revelation?) we are about to attend, to become too general, we will allow but four of our manageresses to express the convened opinion, taking care, however, that the marked peculiarities both in person and opinion, of the genus mistress be faithfully portrayed. Of course the burden of the matter is complainful, for the satisfied ones, if so be such are present, are silent.

As soon as the discussion of the Help Question is in order, some one solicitously inquires of Madam A. as to the relations now existing between herself and help. Now Mrs. A.'s powder is not of the slow kind—it goes off with the merest possible spark, and makes a fuss when it detonates. "My girl isn't worth her salt!" and this explosion settles the status of *one* "help" instanter, so far as lady A.'s influence goes.

Now, in defence of said individual "help," perhaps it is well for us to understand that Madam A. has a few peculiarities—a trait or two by no means bad, in fact highly commendable at times and upon occasions, yet scarcely productive of smooth sailing in the kitchen.—She is one who knows exactly what is and ought to be done in a well regulated household—was brought up to housekeeping—took to it naturally—can't be deceived by scrimp work by any domestic living—in fact, knows, in all seriousness, too much of housekeeping for her own peace of mind. Besides *knowing* it all she is dissatisfied with *doing* less than all, unless some one who can turn off work equal to her own, becomes her culinary assistant. Hence, one less competent than herself is characterized by the aforementioned saline metaphor.

Now, may not Mrs. A.'s girl be a good girl as servant girls go—honest, clean, tidy, ready and willing to work, anxious to please—one whom some might deem a prize? But because, forsooth, she is below her mistress' standard of what a perfect housekeeper should be, she stands before the convention as an indifferent help—one not to be recommended.

That Madame A. confines her remarks to the above expression of doubtful excellence, no one will for a moment infer. Her opinion, however verbosely uttered, simply culminates therein, and Madame B., our next speaker, details *her* domestic grievance.

Her help is "awful shiftless"—can't go ahead and do—has no management—about as good as none at all.

Mrs. B., too, has her peculiarity—that of being a thorough housekeeper theoretically, but with no practical ideas whatever upon the subject. She prefers to sit in her parlor—be bored with no charge of the household—give the girl a long tether, until some culinary feat clashes with her fine theories, and then we prefer to be absent when madam's thundercloud breaks over the poor domestic's head.

Here the fault is evidently on madam's side, but she can't be made to see it, and maid number two is consigned to the list of inefficient help.

Of a widely different class of mistress is Madam C. She is ignorant of the art of housekeeping—never had it to do when younger—has no taste in that direction, yet supposes the possession of a house involves a knowledge of its management. Feeling that her help is her superior in household matters, it chafes her, and drives her into what we men would call bullying her maid. When ignorance, raised to power, begins to dictate to wisdom, the result is imaginable. To say that such a household lacks harmony is dealing tenderly with words. That such a mistress should characterize her maid as presuming—set in her way—an impudent hussy—a pert minx—is not to be wondered at, and the girl that could be otherwise must be a saint.

The most pitiable of mistresses, however, is that class of which Mrs. D. is the representative; not a large number, but a very respectable minority. Mrs. D. literally knows nothing of housekeeping, and will not learn; she is perfectly willing to give *carte blanche* to her hired servant, and also perfectly willing to abide the event, be it what it may. She sees no defects, nor cares to, if only the dreadful business is kept off her hands. Her punishment comes only through others—her husband, her neighbor, her friend—who, seeing defects she will not note, make a point thereof, to the detriment of the maid, of course. Company, callers, visitors, see Madam D.'s difficulty, but common courtesy will not allow them to lay the fault at her door. Is a maid with only a nominal mistress to blame if she cooks and cleans and dresses after her own model, when she is provided with no better?

A remarkable fact is this; not one of these four will praise a servant, however good that servant may be. They make nine-tenths of the inefficient help, and then depreciate their own creations. I do not pretend to argue that all, or even a moiety of our servants are really qualified for their positions, but I do say that they are far above the standard at which they are rated by housekepers generally.

Then, too, any of the above mentioned mistresses, save perhaps the last, may combine with her other faults some one or more of the following drawbacks to kitchen harmony.

LOQUACITY—Constantly talking with a servant, either upon her own duties or other matters. This brings about a degree of familiarity between mistress and maid which is not only inconsistent with their relative positions, but subjects the former to continual mortifications, besides lessening the effect of speech when speech becomes necessary.

RETICENCE—Never deigning to address the servant unless absolutely necessary. This gives a coldness to the relationship between mistress and maid which few of the latter can endure submissively.

SCOLDING—A mania with some mistresses, and the greatest spoiler of servants imaginable. An occasional reprimand may be highly proper when circumstances demand it, but this eternal fault-finding is ruinous to anything like household government.

INGRATITUDE—The idea that paid services are never to be thanked—that commendable acts are never to be praised in the servant—that exhibitions of obligation are beneath the dignity of the feminine head of the house. Nothing so quickly quenches the ardor of the servant that strives to please as a lack of appreciation of efforts in that direction.

INCONSISTENCY—Forbidding at one time what is allowed at another; thus exposing to a watchful servant a weakness which ever begets contempt.

SEVERITY—Frowning upon trifling departures from the strictest code of kitchen discipline—requiring more work than reason claims—insisting upon unremitting labor and no recreation—forbidding visits of any sort from the servant's friends—in fine, instituting a species of serfdom revolting to the genius of this age.

WANT OF CHARITY—Finding no excuse for a degree of ignorance pardonable in a class to which means of getting knowledge are rare—berating omissions of a trifling nature—misconstruing acts of pardonable thoughtlessness into the basest of motives.

SUSPICION—Secretly, if not openly accusing "help" of misdemeanors, (if not crimes) the facts in the case of which cannot be substantiated—perhaps unjustly ruining the character or blighting the prospects of an innocent girl, to whom respectability is as dear as it is to her calumniator, and whose moral decline may date from that thoughtlessly expressed suspicion.

STINGINESS—Grudgingly doling out wages long after falling due—impressing the recipient with a generosity that is but duty, and a mean duty at that — watchfully scrutinizing each mouthful at table and mentally ciphering up the cost thereof.

IMPATIENCE—Expecting immediate compliance with commands which every whim imposes, and repeating such commands again and again before compliance is possible—hurrying a servant from one uncompleted task to another, and distracting her by a multitude of duties not immediately pressing.

Heaping order upon order to an indefinite extent, is too common a fault with otherwise good mistresses. The following is scarcely an exaggeration: Time, five o'clock in the morning. Mistress at servant's door:—"Bridget! Bridget, I say! Get up! Here it is, going on six o'clock! Hurry down and put the kettle on—then make my bedroom fire—now hurry! for you must go to market and get a steak for breakfast—mind, a tender one! Be sure and not forget to stop for the milk—that pumpkin must be put to stew right off, and you must get your pies out of the way before ten o'clock, as the cleaner is coming to-day and you must help her. Remember the walk and porch must be swept before breakfast—the door-plate and knob look fearfully dull—haven't been cleaned since last week and then 't wasn't half done—better scour it—then come and get your orders for groceries so you can go for them while we're at breakfast—and stop at Mrs. Gadditt's on the way, and tell her the sewing circle meets at Mrs. Fitznoodle's this afternoon at two precisely—and call at Mrs. Borrowitt's and tell her I'd like that pattern of mine—the short gored puffed upper skirt I lent her last week—and—well, I'll tell you the rest when I get down stairs!"

Now, I ask, can our dear housekeepers ever expect to have good servants under a *regime* like the present? Can't you guess where the trouble really lies? Do men have such trouble with their employees? If one man hires another, he is generally well served, and both are satisfied—one with the work, the other with the wages.

Must we then confess that women do not understand governing servants? I fear we cannot do otherwise, and until they learn, we may always expect this talk and tirade against them. There *are* good mistresses—those who know just how to deal with their help—who go upon the male principle, viz: assign work reasonably and expect reasonable, and not superhuman results. As a rule, good mistresses make good servants, and I fear that however numerous our inefficient servants are, our unreasonable, impracticable, absurd mistresses are even more so.

Hence, I never hear a company of ladies berating their help, but what I suspect that the fault lies not wholly with the servant—that in the household economy of the mistress there is, somewhere lurking, a secret solution of the difficulty, perhaps even unknown to, but probably too well known by, its miserable possessor.

In a future article I purpose to view the help question from the opposite stand-point—the side of the domestic herself—if, haply, some cogent argument may not be drawn therefrom in support of this, our theory, to wit: that to reform the present system of hired female help, a revolution must take place in the matrons, their mistresses—in the mothers, wives, sisters and daughters—in their methods of instructing, commanding, treating and paying their maids-of-all-work.

---

AT a New Hampshire Teachers' Institute, lately, Professor Cruttenden, of New York, took strong ground against the premature development of the memorizing faculty, and affirmed his belief that "mental arithmetics" killed off more children than did any of the diseases of childhood.

---

ONE manufactory in California uses 1,000 bushels of castor beans per day to make castor oil. This will be pleasant news to children, who are so fond of it.

